# Advice on the Management of the Teeth

**Published:** 1847-10

**Authors:** 


*Advice on the Management of the Teeth.* By T. B. Hamlin, D. D. S. Fellow of the American Society of Dental Surgeons, &c. 12mo, pp. 24, Nashville, Tenn. 1847.

The above is the title of another small popular treatise on the teeth, containing much useful and valuable advice. It is well written, and beautifully gotten up, several pages of it are devoted to the hygiene of the mouth, embodying much useful information upon the subject. The means for the preservation of the health of the dental apparatus are briefly but very clearly pointed out.

The most common of the diseases of the teeth and gums, and their remedial indications, are treated of in a familiar manner, as also artificial teeth, &c.

We have always been an advocate for the dissemination of correct information upon the hygiene of the dental organism, and we should be glad to have such information placed within the reach of every individual, believing as we do, that much good would result from it.—*Balt. Ed.*

*Amblre's Dental Register* for 1847.—We received some months since, from the author, Mr. J. G. Ambler, surgeon dentist of New York, a very neatly gotten-up register, so arranged, that the operations for every day in the year, may be recorded, with such remarks as the peculiarity of each individual case may suggest. Every practitioner should be provided with a case-book of this sort, and if he would accustom himself to note the peculiarities of every case, he would soon collect a large amount of valuable statistical information.

We should have acknowledged the receipt of the above work, and for which we now beg to tender our thanks, at a much earlier period, but for the fact, that soon after receiving it, we were compelled, on account of ill health to leave the city, and it was during our absence that the March number of the Journal was issued; and in preparing the matter for the June number, the Register having been placed in the Infirmary of the Baltimore College of Dental Surgery, for the purpose of recording the cases that were treated there, it entirely escaped our attention.—*Balt. Ed.*

